# Severe maternal morbidity among migrants with insecure residency status in Sweden 2000–2014: a population-based cohort study

**DOI:** 10.1016/j.jmh.2020.100006

**Published:** 2020-11-21

**Authors:** Can Liu, Elizabeth Wall-Wieler, Marcelo Urquia, Suzan L. Carmichael, Olof Stephansson

**Affiliations:** a Division of Neonatal and Developmental Medicine, Department of Pediatrics, Stanford University School of Medicine, Stanford, CA, USA; b Clinical Epidemiology Division, Department of Medicine, Solna, Karolinska Institutet, Stockholm, Sweden; c Manitoba Centre for Health Policy, Department of Community Health Sciences, Rady Faculty of Health Sciences, University of Manitoba, Winnipeg, MB, Canada; d Division of Maternal- Fetal Medicine, Department of Obstetrics and Gynecology, Stanford University School of Medicine, Stanford, CA, USA; e Department of Women's Health, Karolinska University Hospital, Stockholm, Sweden

**Keywords:** Migrant, Residency status, Severe maternal morbidity, Preeclampsia

## Abstract

**Background:**

Migrants with insecure residency status (i.e., undocumented migrants and asylum-seekers, who are denied or waiting for authorized residency) often experience social and psychosocial adversities and limited access to health care. Nonetheless, they have not been profiled on the risk of severe maternal morbidity (SMM), a sentinel measure of maternal health and maternity care.

**Methods:**

A cohort study on all births recorded in the Swedish Medical Birth Register from 2000–2014 (*N* = 1,570,472). Lacking a maternal personal identification number was used as an indicator for insecure residency status (1.3% of all births). We used Poisson regression models to estimate risk ratios of SMM in migrant women with insecure residency status compared to the Swedish-born or migrant women with long-term residency, adjusting for the calendar year of birth, maternal age, and parity.

**Results:**

Overall SMM rate among migrant women with insecure residency status was 21.5/1000 and 14.7/1000 among Swedish-born women. Compared to Swedish-born, migrants with insecure residency status had 50% higher risk of overall SMM (adjusted risk ratio (aRR)=1.54 [1.37–1.74]) and over 80% higher risk of SMM excluding transfusion-only cases (aRR=1.88 [1.37–2.57]). When compared to migrant women with long-term residency, migrant women with insecure residency also had a higher risk of SMM (overall SMM aRR=1.42 [1.26,1.61]; SMM excluding transfusion only cases aRR=1.43 [1.04,1.97]), suggesting that insecure residency conferred additional risks of SMM beyond migration.

**Conclusion:**

Migrant women with insecure residency status had increased risk of severe maternal morbidity.

## Highlights

• Population health of migrants with insecure residency remains largely unknown.
• It is unclear if women with insecure residency are more likely to die from childbirth.
• They showed apparent lower risks of diagnosing pregnancy-related complications in Swedish national birth hospitalization data.
• However, women with insecure residency status were more likely to develop potentially life-threatening conditions.
• Such conditions may be preventable by access to better maternity care

## Introduction

Immigration to Europe has been steadily increasing (World Health Organization, 2018). Among all migrants, asylum seekers live in uncertainty and experience adverse social and psychosocial environments such as lower socioeconomic status, discrimination, and less access to health care (World Health Organization, 2018). Pregnant women in disadvantaged social positions are known to have an increased risk of maternal morbidities, including preeclampsia (Silva et al., 2008), prenatal and postpartum depression (Goyal et al., 2010), and severe morbidities such as eclampsia (Lindquist et al., 2013) or progress into death (Creanga et al., 2012, Kayem et al., 2011).

Undocumented migrants are also in similarly disadvantaged social positions, in addition to experiencing the stress of breaking the law and fear of deportation (Urquia et al., 2015, De Vito et al., 2015). Migrant women with insecure residency status (IRS)^1^—including undocumented migrants and asylum-seekers—have a lower level of well-being during pregnancy (Liu et al., 2019). Nonetheless, they also showed reduced risks of preeclampsia and postpartum hemorrhage (Liu et al., 2019). Given the discrepancy between subjective health and the selected maternal morbidities, maternal health of women with IRS remains unclear.

Examining more severe maternal outcomes in association with IRS will help to illuminate the health and needs of women with IRS. Severe maternal morbidity (SMM) is a composite of potentially life-threatening conditions during pregnancy and childbirth (Callaghan et al., 2012). As a sentinel measure of maternal health and health care, SMM affects approximately 0.5–1.5% of women giving birth and is approximately 50–100 times more common than maternal mortality. Using a national birth register from Sweden, we aim to examine the risk of SMM in women with IRS.

## Methods

**Fig. 1:**
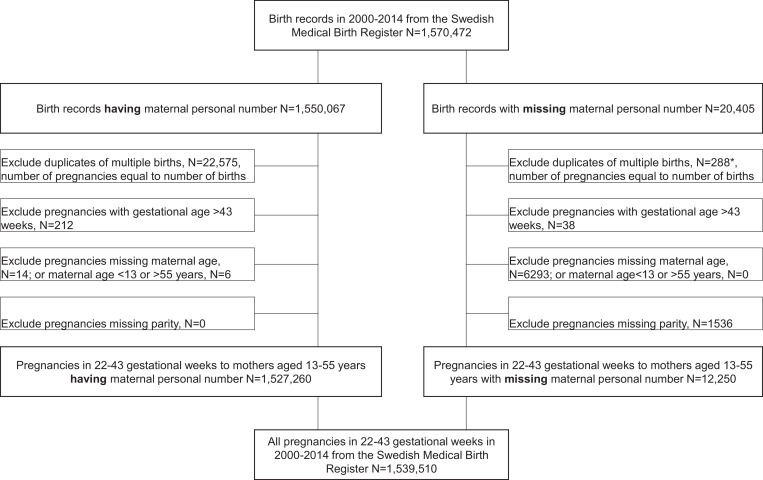
Flowchart of the study population. *Maternal personal identification number is unavailable for linking multiple births, so we used the combination of hospital, birth date and gestational age as linkage between multiple births of the same woman in one birth hospitalization.

We used data of all 1570,472 births (both live and stillborn) recorded in the Swedish Medical Birth Register (MBR) from 2000–2014, of which 20,405 (1.3%) births had no maternal personal identification number (PIN) (Ludvigsson et al., 2009) (Fig. 1). Having a maternal PIN is the default for all persons with a legal residency of 13 months or longer in Sweden. Thus, having a missing PIN can be used as a proxy for not having legal residency, referred to as having IRS. Duplicates of multiple births (*N* = 22,863) were excluded to create the analytical sample of all pregnancies (that ended with a birth). We then excluded gestational age ≥43 weeks when post-terms would have been induced (*N* = 250), and those missing or outlying (*N* = 7849) on the adjustment variables: maternal age (<13 or >55 years), parity, and calendar year.

SMM was defined using an index developed by the United States Centers for Disease Control and Prevention (Callaghan et al., 2012). The index was coded using the Swedish version of ICD-10 codes and modified in adaptation to Swedish clinical practices (Table A.1). SMM cases indicated by blood transfusion alone may include some false negatives since we did not have information on the volume of blood transfusion (Main et al., 2016). Therefore we present SMM with and without transfusion-only cases to provide a range of the SMM risk estimation. Additionally, we examined the distributions of maternal body mass index (BMI) and smoking status measured in early pregnancy, stillbirth, multiple gestation, and mode of delivery.

We used Poisson regression with robust estimation of standard errors to estimate the risk ratios (RR) of SMM of the two migrant groups compared to the Swedish-born group, adjusting for the calendar year of birth, maternal age, and parity. We did not adjust for the other covariates because they are on the causal pathway from migration status to SMM or because their temporality relative to SMM is uncertain. We also compared migrants with IRS to migrants with long-term residency status (LTRS, i.e., foreign-born and had a PIN) to determine the excess risk conferred by IRS beyond the baseline risk of being an immigrant. Also, we explored each groups' top-10 indicators for SMM cases (excluding transfusion only cases). We did not perform statistical tests on the specific and often rare indicators due to the increased probability of having a significant result in multiple tests (type II error).

For sensitivity analysis, we examined SMM risk including the pregnancies missing covariates, which mostly did not have a PIN and were therefore expected to be migrants with IRS. To verify the finding from the previous Swedish study using a different data source (Liu et al., 2019), we additionally compared risks of postpartum hemorrhage (ICD-10 code O72 or O67.8) and preeclampsia (ICD-10 code O14 or O15) among migrants with IRS to Swedish-born women.

All statistical analyses were performed in STATA IC 16.1. The regional ethical review board approved the study in Stockholm (2008/1182-31/4).

## Results

The analytical sample (*N* = 1539,510 pregnancies) comprised 1199,088 (77.8%) Swedish-born, 328,172 (21.3%) migrants with LTRS, and 12,250 (0.8%) migrants with IRS. There were more migrants with IRS in 2000–2004 than in later years, whereas migrants with LTRS steadily increased over time (Table 1). The group with IRS included more women who were younger than 20 years of age, primiparous, multiparous with more than three children or smoking at early pregnancy. Migrants with IRS also had more preterm births, post-term births, stillbirths, and spontaneous vaginal births, but less planned Cesarean births. BMI was missing in 20% of migrants with IRS, compared to about 9% of the other two groups (Table A.2).

**Table 1:** Relative risks of SMM among migrant women with long-term residency and migrant women with insecure residency status in comparison to Swedish-born women (*N* = 1539,510).

	SMM Risk		Relative Risk (95% CI)			
	number of SMM cases/number of pregnancies	SMM per 1000 pregnancies [95%CI]	Compare the two migrant groups to the Swedish-born women		Compare migrant women with insecure residency to migrant women with long-term residency	
			Unadjusted	Adjusted^a^	Unadjusted	Adjusted^a^
SMM Including Blood Transfusion Only Cases						
Swedish-born women	17,685/1199,088	14.7 [14.5–15.0]	1.00 (Reference)	1.00 (Reference)	N/A	N/A
Migrant women with long-term residency	5077/328,172	15.5 [15.0–15.9]	1.05 (1.02,1.08)	1.11 (1.08,1.14)	1.00 (Reference)	1.00 (Reference)
Migrant women with insecure residency	262/12,250	21.4 [18.9–24.1]	1.45 (1.29,1.64)	1.54 (1.37,1.74)	1.38 (1.22,1.56)	1.42 (1.26,1.61)
SMM Excluding 19,963 Blood Transfusion Only Cases						
Swedish-born women	2189/1183,592	1.8 [1.8–1.9]	1.00 (Reference)	1.00 (Reference)	N/A	N/A
Migrant women with long-term residency	832/323,927	2.6 [2.4–2.7]	1.39 (1.28,1.50)	1.40 (1.29,1.52)	1.00 (Reference)	1.00 (Reference)
Migrant women with insecure residency	40/12,028	3.3 [2.4–4.5]	1.80 (1.32,2.46)	1.88 (1.37,2.57)	1.29 (0.94,1.78)	1.43 (1.04,1.97)

a Adjusted for maternal age, parity, and calendar year of birth.

Table 1 shows that migrants with IRS had the highest SMM rates among the three groups. Adjusting for maternal age, parity, and calendar year of birth, migrants with IRS had a 50% higher risk of SMM (adjusted risk ratio aRR= 1.54, 95%CI [1.37,1.74]) than Swedish-born women. The group of migrants with LTRS had a marginally higher risk of SMM than the Swedish-born women. The two migrant groups' SMM risks compared with Swedish-born women were more pronounced when examining SMM excluding transfusion only cases (migrants with IRS aRR=1.88 [1.37,2.57]; migrants with LTRS aRR=1.40 [1.29,1.52]). Compared to migrants with LTRS, migrants with IRS still showed an increased risk of SMM (overall SMM aRR=1.42 [1.26,1.61]; transfusion only excluded SMM aRR=1.43 [1.04,1.97]).

**Fig. 2:**
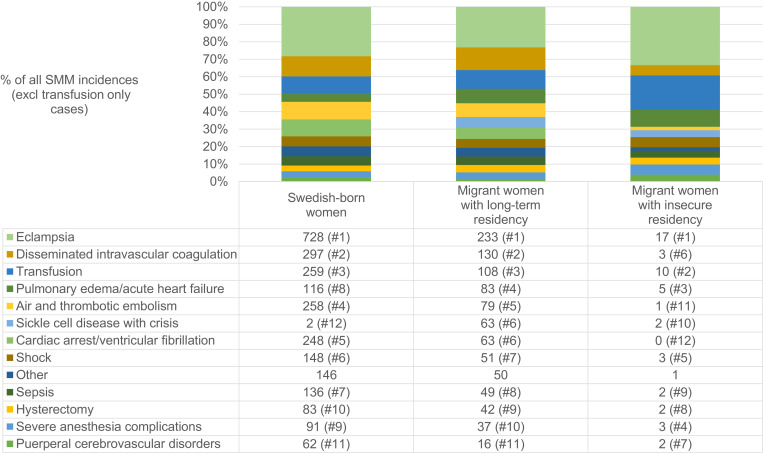
Number and ranking of SMM diagnoses* (excluding transfusion only cases). *Listing the top 12 diagnoses (the union of the top-10 in each group). Column sums may add to more than the total number of cases because each SMM case can have more than one SMM diagnosis. Swedish-born women: 2574 diagnoses in 2189 cases; Migrant women with long-term residency: 1004 diagnoses in 832 cases; Unauthorized migrants: 51 diagnoses in 40 cases.

Fig. 2 shows the number of SMM diagnoses for the top-10 most common SMM indicators in each group (after excluding transfusion-only cases). Eclampsia, pulmonary edema/acute heart failure, and transfusion (co-occurring with other SMM indicators) appeared to be disproportionally common among migrants with IRS. Sickle cell disease with crisis was more common in both migrant groups than in the Swedish-born women.

Sensitivity analysis including the pregnancies missing covariates still showed higher unadjusted risks of SMM than the Swedish-born women (including transfusion-only cases: RR=1.23, 95%CI [1.11–1.37]; excluding transfusion-only cases: RR=1.86, 95%CI [1.46,2.37]), as well as compared to the migrants with LTRS (including transfusion-only cases: RR=1.17, 95%CI [1.06–1.31]; excluding transfusion-only cases: RR=1.34, 95%CI [1.04,1.71]). Migrants with IRS showed lower risks of postpartum hemorrhage (aRR=0.90 [0.84,0.97]) and preeclampsia (aRR=0.68 [0.60,0.77]) than the Swedish-born women.

## Discussion

Migrant women with IRS had a 50% higher risk of SMM than Swedish-born and 40% higher risk than migrant women with LTRS in Sweden. Their SMM risk was further pronounced when excluding transfusion only cases (88% higher than Swedish-born and 40% higher than migrant women with LTRS).

Despite the apparent lower risks of postpartum hemorrhage and preeclampsia, migrants with IRS showed an increased risk of progressing into SMM. This finding is consistent with the poor self-rated health of migrants with IRS (Liu et al., 2019), suggesting migrants with IRS have poor maternal health.

Teasing apart migration-related factors (e.g., language barriers), comparing migrants with IRS to LTRS showed that IRS per se has an impact on maternal health. Given the adverse social, psychosocial, and health care barriers (Urquia et al., 2015, Liu et al., 2019, Keygnaert et al., 2016), preventing SMM may have been particularly challenging in migrants with IRS. Their contradicting low risk of preeclampsia versus the high risk of eclampsia also suggested inadequate antenatal care, as previously shown (Liu et al., 2019).

Barriers to accessing preventative care may affect their health and health care use. Even within a universal health care system, there were legal barriers for these women to access maternity care early, especially for undocumented migrants during the study period (2000–2014). In 2008, the universal health care system of Sweden started to provide free emergency care to all persons, regardless of residency. However, universal access to free non-emergency maternity care (e.g., antenatal care) only began in 2013 (Sveriges Riksdag, 2008, Sveriges Riksdag, 2013). The accessibility to maternity care for migrants with IRS in this study still approximates situations in many other countries today (Keygnaert et al., 2016).

Besides care-related factors, their health status before pregnancy and teenage pregnancy rate require attentions. Although rare, sickle cell disease risk deserves special care for migrants from regions with historical selection pressure on malaria resistance gene (Allison, 1954). In addition to SMM, their increased risk of preterm birth also raises the concern for the dual burden of SMM and preterm birth (Lyndon et al., 2019).

This study has several implications for health policies. Same as maternal deaths, most SMMs are potentially preventable through adequate health care (Lawton et al., 2014). Limited access to antenatal care for migrants with IRS probably contributed to delayed diagnoses of preeclampsia and progressions into eclampsia. IRS group's higher missing rate of BMI also implies delayed first antenatal visits. To end preventable deaths of women—every woman must have access to antenatal care (Every Woman Every Child, 2015). With inequities exacerbated during the ongoing global pandemic, governments should urgently act to ensure basic health care services such as antenatal care, for all women, irrespective of legal residence status (World Health Organization 2020).

In addition, our findings showed an increased risk of teenage pregnancy in migrants with IRS. The lack of health care access, including family planning services, may increase the risk of having unplanned pregnancies among the migrants with IRS. In particular, undocumented migrants lacked education opportunities before 2013 in Sweden (Bunar, 2017), which could contribute to increased risk of pregnancies in adolescence or young adulthood (Girma and Paton, 2015). Thus, the increased teenage pregnancy rate in migrants with IRS has two implications. First, national policies are needed to protect every woman's fundamental human right to control her own reproduction. Second, migrant children with IRS should have access to education.

The study also showed an overlooked issue in population health research using administrative data. We notice that register-based studies mostly do not cover migrants with IRS while claiming national representativeness (Gauffin, 2020). The adverse pregnancy outcomes of migrants with IRS should draw more attention, especially for countries that have a larger migrant population with IRS, e.g. 6.9–7.4% of all births in the US are estimated to be born to women with undocumented migration status (Camarota et al., 2018, Passel and Cohn, 2016). International comparative studies need to consider the proportions of migrants with IRS represented in different administrative data.

The study has several strengths. We took advantage of PIN (Ludvigsson et al., 2009) usage in the Swedish health care system as a measure of residency status at birth hospitalization. The lack of routinely collected data on migrants with IRS (particularly undocumented migrants) is universal. This study adds to the few population-based studies to examine the health of this migrant group (De Vito et al., 2015). Provided the proxy indicator from administrative data, we examined almost all birth hospitalizations of migrants with IRS. Based on the large dataset and the rich information from the MBR, we were able to measure the rare outcome of SMM from ICD-10 codes following an internationally recognized definition.

There are some limitations in taking this approach to measure IRS. It remains possible that migrants with IRS include women who were neither asylum-seekers nor undocumented migrants, such as tourists or short-term visitors who have better socioeconomic and health status. Nevertheless, we expect such women to be a small group. Almost all women with IRS did not have registered information on their region of origin, which prevented further comparison with migrants with LTRS. Undocumented migrants might fear being reported thus did not provide personal information to the health care system, which made them more likely to be excluded due to missing data. Besides, it was impossible to differentiate undocumented migrants from asylum-seekers. Further information on sociodemographics (e.g. education) would also be helpful to our study but were not available, due to limited linkages across registers for the IRS group.

## Conclusion

Compared to Swedish-born or migrants with long-term residency, migrant women with insecure residency status were more likely to have life-threatening conditions during childbirth.

## Authors' contributions

CL conceived the study, analyzed the data, drafted the first manuscript. EWW helped writing the first manuscript. MU provided literature background. SLC supervised and directed the study implementation. OS obtained data and provided background knowledge for using the data. EWW, MU, SLC and OS interpreted the results and provided significant feedback on the drafts. All authors reviewed and approved the final manuscript.

## Data availability statement

The National Board of Health and Welfare in Sweden approved the request and provided the anonymous data after the ethical approval was obtained from the Regional Ethics Review Board. The data can be requested from the National Board of Health and Welfare in Sweden (https://www.socialstyrelsen.se/). The raw data cannot be shared in a public repository based on data use agreement.

## Declaration of Competing Interest

The authors report no competing interest.

## Figures and Tables

**Table A.1:** ICD-10 codes of United States Centers for Disease Control and Prevention SMM index and the Swedish adaptation.

Severe Maternal Morbidity Indicator	International ICD-10 codes	Translation to Swedish version ICD-10 codes	Modification on Swedish version based on clinical expert knowledge
1. Acute myocardial infarction	I21.xx, I22.x	I21x, I22x	
2. Aneurysm*	I71.xx	I71x, I79x	
3. Acute renal failure	N17.x, O90.4	N17x, O904	
4. Adult respiratory distress syndrome	J80, J95.1, J95.2, J95.3, J95.82x, J96.0x, J96.2x R09.2	J80, J951, J952, J953, J958, J960, J969, R092	
5. Amniotic fluid embolism	O88.1x	O881	
6. Cardiac arrest/ventricular fibrillation*	I46.x, I49.0x	I46x, I49x	
7. Conversion of cardiac rhythm	5A2204Z, 5A12012	DF010, DF012	Add temporary code procedure codes V9216, V9203
8. Disseminated intravascular coagulation	D65, D68.8, D68.9, O72.3	D65x, D688, D689, O723	Remove D688, D689 (unspecific DIC with unknown diagnosing practice in Sweden)
9. Eclampsia	O15. X	O15x	
10. Heart failure/arrest during surgery or procedure	I97.12x, I97.13x, I97.710, I97.711	I97x	
11. Puerperal cerebrovascular disorders	I60.xx- I68.xx, O22.51, O22.52, O22.53, I97.81x, I97.82x, O87.3	I60x, I68x, O225, I978, O873	
12. Pulmonary edema / Acute heart failure	J81.0, I50.1, I50.20, I50.21, I50.23, I50.30, I50.31, I50.33, I50.40, I50.41, I50.43, I50.9	J81x, I50x	
13. Severe anesthesia complications	O74.0, O74.1, O74.2, O74.3, O89.0x, O89.1, O89.2	O740, O741, O742, O743, O890, O891, O892	
14. Sepsis	O85, O86.04, T80.211A, T81.4XXA, T81.44xx, R65.20, A40.x, A41.x, A32.7	O85x, T802, T814, A40x, A41x, A427, A327, R652	Remove ICD-10 code O85x (not used specifically for Sepsis in Sweden)
15. Shock	O75.1, R57.x, R65.21, T78.2XXA, T88.2 XXA, T88.6 XXA, T81.10XA, T81.11XA, T81.19XA	O751, R57x, R65x, T782, T882, T886, T811	
16. Sickle cell disease with crisis	D57.0x, D57.21x, D57.41x, D57.81x	D57x	
17. Air and thrombotic embolism	I26.x, O88.0x, O88.2x, O88.3x, O88.8x	I26x, O88x	
18. Blood products transfusion	30,233 Peripheral vein, percutaneous	DR029	Add temporary procedure codes VPA00, VPA02, VPA04, VPA06, VPA07, V9209
19. Hysterectomy	0UT90ZZ, 0UT94ZZ, 0UT97ZZ, 0UT98ZZ, 0UT9FZZ	LCD00, LCD01, LCD04, LCD10, LCD11, LCD96, LCD97	
20. Temporary tracheostomy	0B110Z, 0B110F, 0B113, 0B114	GBB00, GBB03, GBB96	Add temporary procedure code V9122
21. Ventilation	5A1935Z, 5A1945Z, 5A1955Z	DG002	Add temporary procedure code V9128

**Table A.2:** Characteristics of women by migration status.

	Swedish-born women	Migrant women with long-term residency	Migrant women with insecure residency
	*N* = 1199,088	*N* = 328,172	*N* = 12,250
	n (Col %)	n (Col %)	n (Col %)
Calendar year of birth			
2000–2004	379,018 (31.61)	82,116 (25.02)	4676 (38.17)
2005–2009	407,243 (33.96)	107,691 (32.82)	3333 (27.21)
2010–2014	412,827 (34.43)	138,365 (42.16)	4241 (34.62)
Age(years)			
13–19	17,628 (1.47)	6488 (1.98)	1013 (8.27)
20–29	503,262 (41.97)	150,412 (45.83)	7336 (59.89)
30–39	638,277 (53.23)	156,803 (47.78)	3629 (29.62)
40–49	39,876 (3.33)	14,404 (4.39)	272 (2.22)
50–55	45 (0.00)	65 (0.02)	0 (0.00)
Parity			
1	541,844 (45.19)	132,650 (40.42)	5828 (47.58)
2–3	602,657 (50.26)	160,225 (48.82)	5059 (41.30)
4 or more	54,587 (4.55)	35,297 (10.76)	1363 (11.13)
BMI categories			
Underweight	22,193 (1.85)	10,559 (3.22)	316 (2.58)
Normal weight	670,733 (55.94)	168,816 (51.44)	4937 (40.30)
Overweight	270,755 (22.58)	82,564 (25.16)	3103 (25.33)
Obesity Class I	88,591 (7.39)	27,964 (8.52)	1084 (8.85)
Obesity Class II	38,732 (3.23)	9727 (2.96)	351 (2.87)
Missing	108,084 (9.01)	28,542 (8.70)	2459 (20.07)
Smoking in early pregnancy			
No smoking	1047,615 (87.37)	291,756 (88.9)	10,592 (86.47)
1–9 cig/day	67,455 (5.63)	16,756 (5.11)	803 (6.56)
10+ cig/day	23,201 (1.93)	4978 (1.52)	317 (2.59)
Missing	60,817 (5.07)	14,682 (4.47)	538 (4.39)
Multiple birth	17,856 (1.49)	4425 (1.35)	179 (1.46)
Gestational age in completed weeks			
Very preterm 22–31	10,103 (0.84)	3364 (1.03)	140 (1.14)
Moderate preterm 32–36	56,332 (4.70)	14,801 (4.51)	754 (6.16)
Term 37–41	1047,692 (87.37)	288,407 (87.88)	10,257 (83.73)
Post-term 42–44	84,338 (7.03)	21,449 (6.54)	1051 (8.58)
Missing	623 (0.05)	151 (0.05)	48 (0.39)
Stillbirth	3940 (0.33)	1503 (0.46)	63 (0.51)
Mode of delivery			
Spontaneous vaginal	906,060 (75.56)	248,582 (75.75)	9482 (77.40)
Instrumental vaginal	91,205 (7.61)	22,723 (6.92)	818 (6.68)
Planned Caesarean	102,331 (8.53)	27,669 (8.43)	870 (7.10)
Emergency Caesarean	94,926 (7.92)	28,067 (8.55)	1002 (8.18)
Missing	4566 (0.38)	1131 (0.34)	78 (0.64)

## Footnotes

1 IRS: Insecure residency status; LTRS: long-term residency status; MBR: Swedish Medical Birth Register; SMM: Severe maternal morbidity; PIN: personal identification number.
